# Inhibition of Glyoxalase-I Leads to Reduced Proliferation, Migration and Colony Formation, and Enhanced Susceptibility to Sorafenib in Hepatocellular Carcinoma

**DOI:** 10.3389/fonc.2019.00785

**Published:** 2019-08-20

**Authors:** Maurice Michel, Marcus Hollenbach, Sabine Pohl, Cristina Ripoll, Alexander Zipprich

**Affiliations:** Laboratory of Molecular Hepatology, Department of Internal Medicine I, Martin Luther University Halle-Wittenberg, Halle (Saale), Germany

**Keywords:** EP, BrBzGSHCp2, proliferation, migration, colony formation

## Abstract

**Background:** Glyoxalase-I (Glo-I) is essential for detoxification of methylglyoxal (MGO), a byproduct of glycolysis. Overexpression of Glo-I has been linked to multi-drug resistance in cancer therapy. The aim of this study was to analyze Glo-I in hepatocellular carcinoma (HCC) and the effect of the multi-tyrosine kinase inhibitor sorafenib on Glo-I.

**Methods:** Expression and specific activity of Glo-I was measured in human HCC samples, HCC-cell lines (HepG2, Huh7) and a hepatocyte cell line (AML 12). Cells were either treated with Glo-I inhibitors, ethyl pyruvate (EP, 1–20 mM) and BrBzGSHCp2 (1–10 μM), or sorafenib (2.5–10 μM) and protein expression (Western Blot), proliferation (WST-assay), migration (scratch assay), and colony formation (clonogenic assay) were assessed.

**Results:** High expression of Glo-I was detected in human HCC tissue samples. Huh7 showed highest expression and activity of Glo-I and revealed highest proliferation compared to AML 12 and HepG2. Targeting Glo-I by EP or BrBzGSHCp_2_ led to significantly reduced proliferation (20 mM EP 24 h: 57 ± 12%), migration and colony formation. Glo-I inhibition by 20 mM EP resulted in reduced expression of PDGFR-β (18 ± 10%), VEGFR2 (46 ± 11%), VEGF (61 ± 10%), pERK/ERK (62 ± 6%), NF-κB (44 ± 12%) as well as stimulation of Nrf2 (243 ± 36%). Similar results were seen with BrBzGSHCp2. Sorafenib treatment revealed elevation of Glo-I (10 μM: 209 ± 25%) and MGO. Co-treatment of EP and sorafenib led to an additional reduction of proliferation compared to sorafenib alone.

**Conclusion:** Glo-I is positively correlated with HCC proliferation. Inhibition of Glo-I reduced proliferation, migration, and colony formation. In turn, sorafenib increases Glo-I. Co-treatment using Glo-I inhibitors could enhance susceptibility of HCC to sorafenib.

## Introduction

Hepatocellular carcinoma (HCC) is the most frequent primary liver cancer and is ranked as the sixth most common neoplasm and the third leading cause of cancer-related death worldwide (1). Despite curable interventions at an early stage of the disease, treatment options in advanced HCC are rare and often respond poorly (2). Up to date only three drugs for medical treatment are approved and comprehensively available. The most frequent used drug, the multi-tyrosine kinase inhibitor sorafenib, has shown beneficial effects in clinical trials of advanced-stage HCC with a median increase in survival of 2.8 months (3). Thus, new approaches and targets for treatment of advanced HCC are urgently needed (4).

In HCC, as in many other cancers, glycolysis is highly upregulated in order to meet elevated energy demands (5), commonly referred to as the Warburg effect (6). However, high glycolytic activity yields toxic by-products, such as the dicarbonyl compound methylglyoxal (MGO). MGO is a highly potent glycating agent and reacts with nucleic acids, proteins and lipids. As a consequence, MGO-derived advanced glycation end products (AGEs) lead to mitochondrial protein dysfunction, enzyme inactivation, mutagenesis, and apoptosis (7). In addition, AGEs bind to their receptor, RAGE, and activate intracellular signaling pathways (8). This so-called “dicarbonyl stress” results in oxidative stress and was shown to be implicated in carcinogenesis (9, 10). In order to prevent intracellular toxic MGO levels, MGO is detoxified by the cytosolic glyoxalase system. Glyoxalase-I (Glo-I) and glyoxalase-II (Glo-II) catalyze the conversion of MGO into unreactive compounds (Supplementary Figure 1) (11).

In the western world, HCC develops most commonly in patients with liver cirrhosis (12). Since HCC is a primary liver cancer there are common pathways activated in cirrhosis and HCC. For example, activation of different inflammatory and transcriptional pathways (IL-1, TNF-α, NF-κB) lead to the development of cirrhosis and HCC (13). Recent data from our lab revealed reduced expression but higher activity of Glo-I in hepatocytes from cirrhotic livers (14). Inhibition of Glo-I in cirrhosis leads to less fibrosis and decreased levels of α-SMA, TGF-β, and NF-κB.

Although, overexpression of Glo-I is known to promote cell migration, proliferation and resistance toward cytotoxic chemotherapy in cancer calls (15), current data on Glo-I in HCC is yet non-cohesive and lacks investigations of the underlying molecular pathways following Glo-I inhibition (16–18). Furthermore, the effect of sorafenib on expression and activity of Glo-I in HCC remains unknown and has not been elucidated so far.

The aim of our study was to (I) investigate the expression and activity of Glo-I in tissue from different HCC patients and differently invasive HCC cell lines. Then (II) analyze the effects of a partial inhibition of Glo-I by two pharmacologic inhibitors on malignancy-associated behavior and pathways as well as (III) to examine the influence of sorafenib on Glo-I activity and expression.

## Materials and Methods

### Tissue Collection

Tissue samples were collected from liver biopsies of HCC patients from the Martin-Luther University Halle-Wittenberg. Informed consent was obtained from all patients before biopsy, and all the procedures were approved by the Medical Ethics Committee of the Martin-Luther University Halle-Wittenberg (2012-5). All tissue samples were fixed in formaldehyde, then embedded in paraffin and stored at room temperature for further analysis. Medical records were evaluated for patient demographics including age, gender, Child-Pugh score, and BCLC-stages as well as grade of varices.

### Immunofluorescence and Determination of Staining Intensity

Sections of paraffin blocks were made with HM 325 (microm, Walldorf, Germany) and dried overnight at room temperature (RT). A descending alcoholic series was followed by demasking and boiling at 250°C. Sections were blocked with 3% H_2_O_2_ for 30 min (pharmacy of the University of Halle) and for 1 h in 5% BSA (Roth, Karlsruhe, Germany) and 0.5% tween (Roth). Primary antibody [Glo-I (mouse monoclonal IgG, MA1-13029, Pierce, Waltham, Massachusetts, USA)] was incubated over night at 4°C. Sections were washed three times with PBS and incubated with secondary antibody [anti-mouse (IgG-HRP, 715035151, donkey origin, Dianova, Hamburg, Germany)] for 1 h at RT. Additional washing was followed by staining with DAB (ImmPACT, Vector, Burlingame, USA) for 2–10 min. The staining reaction was stopped in distilled water. For controls, secondary antibodies lacking primary antibody were incubated. Overview pictures and liver sections were analyzed using the Keyence Biozero BZ 8000 microscope with BZ Viewer (Osaka, Japan). At least 20 sections per biopsy were analyzed. Staining intensity was calculated according to the Quick-Score (Q): Results were scored by multiplying the percentage of positive cells by the intensity (Q = P × I; maximum = 300). Intensity was determined as 1+, 2+, or 3+ according to absent, partial or complete staining (19).

### Cell Culture and Cell Lysates

The HCC-cell lines HepG2 (human) and Huh7 (human), and the hepatocyte cell line AML12 (mouse) were used. HepG2 were cultured in RPMI 1640 (Biochrom, Berlin, Germany), Huh7 in DMEM and AML12 in DMEM-F12 (Gibco Life Technologies, CA, USA), containing 10% heat inactivated FCS and 1% penicillin/streptomycin (P/S). Cell cultures were kept in an incubator at 37°C, 5–10% CO_2_ and the medium was replaced twice per week.

For experiments, cells were seeded in 100 mm dishes (TPP, Trasadingen, Switzerland) at 300.000 cells per dish. At a confluence of 70%, cells were treated with two different inhibitors of Glo-I (20, 21), ethyl pyruvate (EP; 1–20 mM) or BrBzGSHCp_2_ (1–10 μM) (all from (Sigma-Aldrich, Steinheim, Germany), or sorafenib (2.5–10 μM, Bayer Health Care, Leverkusen, Germany) in serum-free medium. After 24 h of incubation, cells were washed twice with PBS (PAA, Pasching, Austria) on ice and lysed with 100 μl RIPA buffer (Sigma-Aldrich). After scraping, cells were centrifuged at 13,000 × g and 4°C for 15 min. Supernatants for enzyme kinetics or Western Blot analysis were collected and stored at −80°C. Protein concentrations were determined using BCA-method following instructions of the manufacturer (Sigma-Aldrich).

For RNA-Analysis, instead of RIPA buffer, 500 μl of trizol (Qiazol, Qiagen, Hilden, Germany) was used. RNA-isolation was adapted according to prior published protocols (22). RNA-concentrations were determined using photometric measurement.

### Western Blot Analysis

Protein lysates were boiled for 5 min at 95°C in SDS protein buffer (Thermo-Scientific, Rockford, USA) and separated by SDS-PAGE following transfer to PVDF membrane. Primary antibodies were Glo-I (1:250; SC-67351), NF-κB (1:500; p65 subunit, SC-372), Nrf2 (1:500; SC-722, all mouse monoclonal AB, all Santa Cruz Biotechnology, Dallas, Texas, USA), p44/42 MAPK (ERK1/2) (1:1000; CST-9102, rabbit polyclonal), phospho-p44/42 MAPK (pERK1/2) (1:2000; CST-4370, rabbit monoclonal), PDGFR-β (1:1000; CST-4564, rabbit monoclonal IgG), VEGFR2 (1:1000; CST-9698, rabbit monoclonal IgG, all Cell Signaling Technology, Boston, Massachusetts, USA), VEGFA (1:500; PA1080, rabbit polyclonal IgG, Booster Biological Technology Co. Ltd, Fremont, California, USA), Vinculin (1:10000; ab129002, rabbit monoclonal IgG, Abcam plc, Cambridge, UK) and GAPDH (1:500; MAB374, mouse monoclonal IgG1). Secondary antibodies were anti-mouse (1:1000; 7076P2 IgG-HRP, horse origin), anti-rabbit (1:1000; 7074P2, IgG-HRP, goat origin, all Cell Signaling Technology, Boston, Massachusetts, USA), and anti-goat (1:1000; 705-035-003, IgG-HRP, donkey origin, Dianova). Western Blot signals were quantified using an imager (Fusion-Fx-7 with BD-Software, Peqlab, Erlangen, Germany). Signals were normalized to their respective loading controls using ImageJ-Software (v. 1.48, http://imagej.nih.gov).

### RT-PCR

Isolated RNA was performed with DNAse for 30 min at 37°C, followed by 10 min at 65°C in stop solution (Promega, Madison, USA). Correct DNA-digestion was checked by control-gel. First-strand cDNA was generated from normalized RNA amounts using Oligo-(dT)-primers and the RevertAid Premium First Strand cDNA Synthesis Kit (Fermentas, Rockford, USA) following instructions of the manufacturer. RT-PCR was performed with taq-polymerase (Red PCR master mix, stratec, Berlin, Germany) and specific primer pairs: GAPDH (1,177 bp fragment; forward: GACCCCTTCATTGACCTC, reverse: GCAATGCCAGCCCCAG; Program: 95°C for 2 min, (95°C for 30 s, 58°C for 45 s, 72°C for 45 s) × 32, 72°C for 2 min), human Glo-I (921 bp fragment; forward: CTTCTGGGGTTTCAATTCCTC, reverse: AATCCATTTCACCCAAAAAGG), mouse Glo-I (940 bp fragment; forward: GATTTGGTCACATTGGGATTG, reverse: AGAGAGCATAGGCCAGACTCC). For Glo-I primer pairs, we used the following PCR program: 95°C for 2 min, (95°C for 30 s, 56°C for 45 s, 72°C for 45 s) × 30, 72°C for 2 min for human primer pairs and 95°C for 2 min, (95°C for 30 s, 60°C for 45 s, 72°C for 45 s) × 30, 72°C for 2 min for mouse primer pairs.

### Measurement of Glo-I Activity

Activity of glyoxalase I (Glo-I, E.C.4.4.1.5) was determined by measurement of the reaction intermediate S-D-lactoylglutathione, with ascending absorbance at 240 nm. Absorbance was measured for 5 min at 25°C in crystal cuvette (Hellma, Berlin, Germany) with a photometer (amersham ultrospec 2100 pro, amershampharmacia biotech, Cambridge, England). For each test, 2 mM GSH (Roth) and 2 mM MGO (Sigma-Aldrich) were incubated for 90 s in 50 mM phosphate-buffer (Na_2_HPO_4_, pH 7.0, Roth), and 10 μl of undiluted cell lysate were used per test. Each probe was measured three times. Phosphate-buffer was set as reference. Enzyme activity was calculated in U by formula: A = (ΔE/min × V)/(ε × d × v). ε for S-D-lactoylglutathione was 2.86 (mol/l × cm). For specific activity, U was referred to protein-concentration (23).

### Proliferation Assay (WST)

Cell proliferation was measured in 96 well plates (5,000 cells/well, TPP) using Colorimetric Cell Viability Kit I (WST-8, Promo Cell, Heidelberg, Germany) following the instructions of the manufacturer. Relative absorbance at 450 nm was measured at time intervals of 6, 12, and 24 h in absence or presence of 1–20 mM EP, 1–10 μM BrBzGSHCp2, or 2.5–10 μM sorafenib.

### Clonogenic Assay/Colony Forming Assay

The influence of EP on the colony-forming behavior of Huh7 cells was determined using 6 well plates (TPP) (24). Cells were seeded at a density of 2,000 cells/well and left overnight. Medium change containing EP (0–20 mM), 10% FBS, and 1% P/S, was conducted daily for 7 days until colonies were detected. After 7 days cells were stained with Coomassie Blue (Applichem, Darmstadt, Germany) according to a standardized staining protocol (25), and images using a commercial scanner were captured for further analysis. For statistical analysis, the Image J plugin “Colony Area” was used (26). In another assay, cells were only treated for 4 or 24 h with different EP concentrations (0–20 mM), and then left untreated in the incubator for 7 days. Statistical analyses were performed by means of at least three independent experiments.

### Wound-Healing Assay

To assess cell migration in Huh7 cells upon treatment with EP, IBIDI μ-dishes with 2 well-inserts (Culture-Insert 2 Well in μ-Dish, 35 mm high, ibiTreat, IBIDI, Heidelberg, Germany) were used as described by protocols (27) and instructions of the manufacturer. Cells were seeded at a density of 300,000 cells and incubated for 24 h until confluency was reached. The insert was then removed with sterile tweezers, while a clear cell-free gap (500 μm) was visible. Medium was removed, and dishes were washed gently with PBS to remove cell debris. Fresh medium containing EP (0–20 mM), 10% FBS, and 1% P/S was added. Pictures of the wound area were taken with a Keyence Biozero BZ 8,000 microscope before the treatment and after certain time intervals at 6, 12, and 24 h. The scratch area was measured with Image J and the “MRI Wound Healing Tool Plugin” (http://dev.mri.cnrs.fr/projects/imagej-macros/wiki/Wound_Healing_Tool). Statistical analyses were performed by means of at least three independent experiments.

### Elisa

For determination of MGO concentrations, 10 μl of protein lysates were used per well-following instructions of the manufacturer (MBS2605842, MyBiosource, San Diego, USA).

### Statistics

Results are expressed as mean ± SD. Comparisons between groups were analyzed by one-way ANOVA, followed by *post-hoc* Bonferroni correction to detect differences between groups. *P* < 0.05 were considered as statistically significant. GraphPad Prism 4.0 software was used.

## Results

### Glo-I Is Highly Expressed in Human HCC Tissue

**Figure 1:**
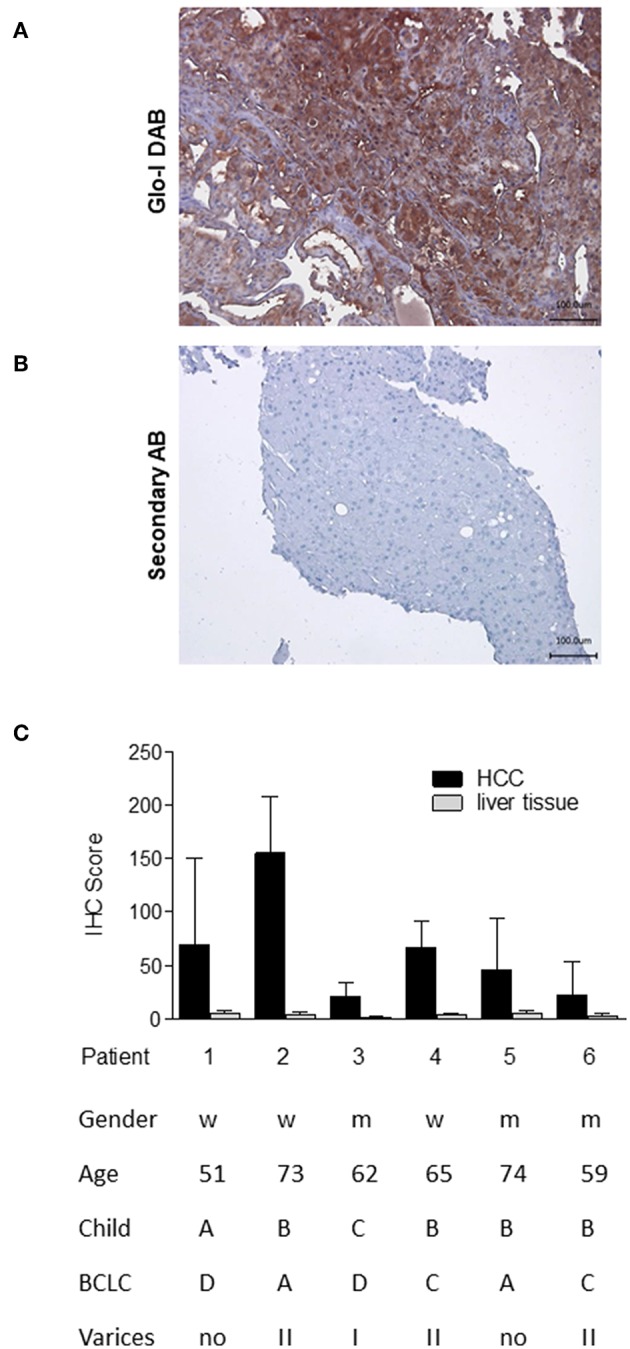
Expression of Glo-I in human HCC obtained by liver biopsy. **(A)** Representative image of Glo-I DAB-immunostaining indicates high expression of Glo-I in HCC biopsies. Staining controls lacking the primary antibody are presented in the lower line. **(B)** Quantification of at least 20 sections per biopsy using the Quick-Score (Q): Q = P [percentage of cells) × I [intensity absent (1), partial (2), complete (3) staining], maximum = 300. Quick-Score was also calculated in non-HCC cirrhotic liver tissue. In all analyzed specimens, Quick-Score of non-HCC tissue was much lower than in HCC tissue **(C)** Clinical data, including Child-Pugh classes and BCLC stages of analyzed patients.

In order to analyze the expression of Glo-I in human HCC, immunohistochemistry of liver biopsies (*n* = 6) was performed and clinical parameters were assessed (Figure 1). Half of the patients were male, and the mean age was 64 years. Patients revealed a wide spectrum of liver diseases stadium, the majority were of Child-Pugh class B (*n* = 4), but also of BCLC stadium A (*n* = 2), C (*n* = 2), and D (*n* = 2). In all patients, cirrhosis was caused by alcohol consumption. Representative images of HCC samples are shown in Figure 1A, with the calculated Quick-Score of Glo-I staining intensity (Figure 1B) verifying Glo-I in all investigated HCC patients, and comparing Glo-I expression in cancerous to non-cancerous tissue. The mean overall Quick-Score was 63.1 ± 64.7. Overall, expression of Glo-I was higher in HCC tissue as compared to non-HCC cirrhotic tissue in all examined specimens.

### Correlation of Glo-I Expression and Specific Activity With Proliferation of HCC Cells

**Figure 2:**
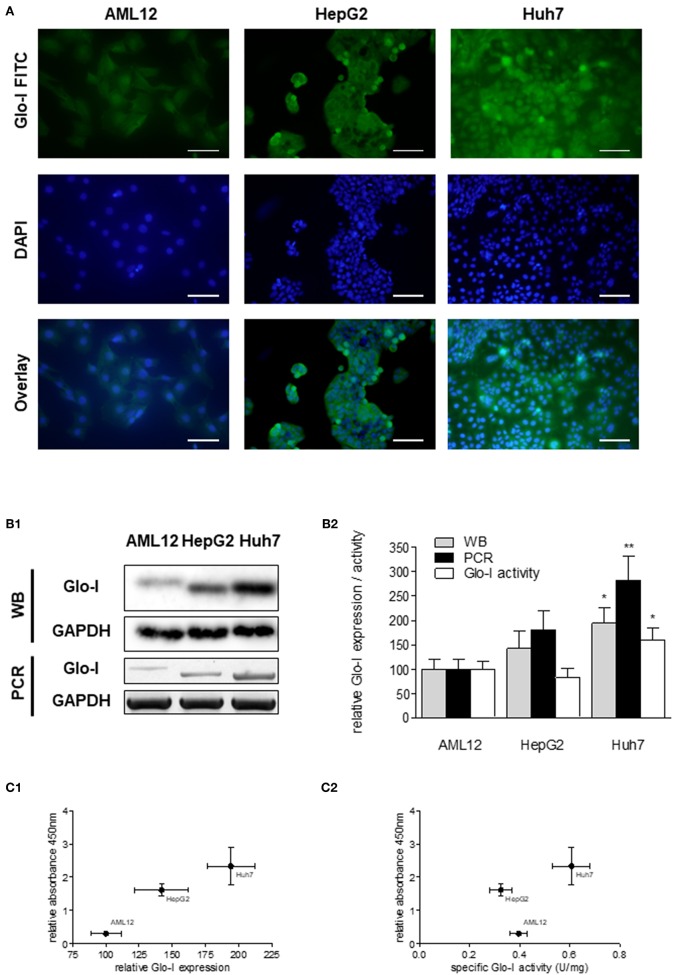
Expression and specific activity of Glo-I in HCC cell lines. **(A)** Immunofluorescence staining of Glo-I in AML12, HepG2, and Huh7 cell lines. Glo-I-FITC signal is shown in green color (upper row), staining of nuclei with DAPI can be found in the middle row. Overlay of Glo-I and DAPI staining (lower row) indicates cytosolic expression in all cell lines, with highest expression in Huh7. Scale bars: 100 μm. **(B1)**, Protein and mRNA analysis of Glo-I expression was compared among the three cell lines by Western Blot and RT-PCR. Quantifications **(B2)** revealed expression of both mRNA and protein level to be highest in Huh7 cell line, as compared to HepG2 and AML12. **(C1)** Correlation of relative Glo-I expression and cell proliferation among AML12, HepG2, and Huh7 indicated highest relation in Huh7. **(C2)** Likewise, correlation of specific enzymatic activity of Glo-I (U/mg) and cell proliferation showed highest relation in Huh7 cell line. Results are represented as mean ± S.D. of at least three independent experiments. *P < 0.05, **P < 0.01.

Next, expression and activity of Glo-I was studied in the HCC cell lines HepG2 and Huh7, and the hepatocyte cell line AML12 by means of Immunofluorescence (Figure 2A), Western Blot, RT-PCR, and enzyme kinetics (Figures 2B1,B2). Protein and mRNA expression of Glo-I was significantly higher in Huh7 [protein (p): 194 ± 31%, *p* < 0.05; mRNA (m): 282 ± 48%, *p* < 0.01], as compared to our control AML12 (*p*: 100 ± 20%, m: 100 ± 21%; Figure 2B2). In fact, Huh7 showed highest mRNA expression among the cell lines studied. In HepG2, mRNA expression (m: 181 ± 39%, *p* < 0.05; Figure 2B2) was significantly higher compared to AML12, whereas no significant difference was seen in protein expression or kinetic activity (HepG2, p: 142 ± 35%, a: 82 ± 19%, *p* > 0.05). In addition, the specific activity of Glo-I was elevated in Huh7 (0.6 ± 0.1 U/mg; 160 ± 26%, *p* < 0.05) and with no difference in HepG2 (0.32 ± 0.1 U/mg, 82 ± 19%, *p* > 0.05) compared to AML12 (0.4 ± 0.1 U/mg, 100 ± 15%). Activity of Glo-I and mRNA expression was significantly higher in Huh7 in contrast to HepG2 (Huh7 a: 160 ± 26%, m: 282 ± 48% vs. HepG2 a: 82 ± 19%, m: 181 ± 39%, all *p* < 0.05).

Expression and specific activity of Glo-I correlated with the proliferative activity of the different cell lines. Proliferation, as indicated by relative absorbance at 450 nm in the WST-assay, was blotted against relative Glo-I expression (Figure 2C1) and specific Glo-I activity (Figure 2C2). The results indicated a positive correlation of Glo-I with cell proliferation as Huh7 showed highest absorbancy at 450 nm.

### Partial Inhibition of Glo-I by EP Decreases Proliferation and Inhibits Related Pathways in Huh7 Cells

**Figure 3:**
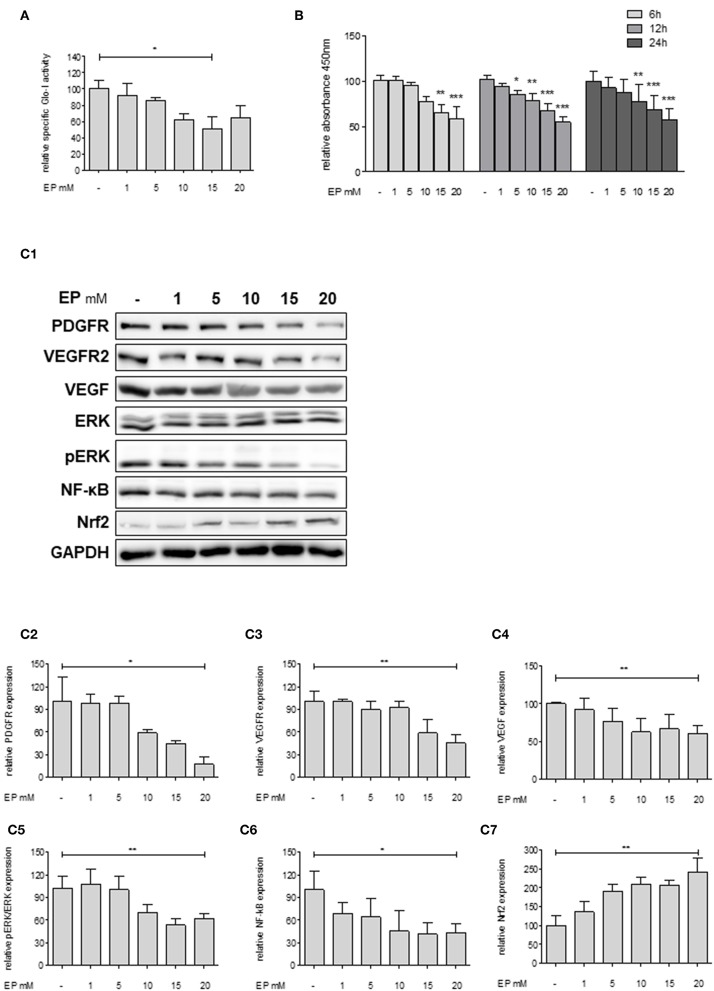
Effect of partial inhibition of Glo-I by EP on proliferation and HCC-related signaling pathways in Huh7 cells. **(A)** Glo-I specific activity was significantly reduced after 24 h EP treatment in a concentration-dependent manner in Huh7 cells. Doses of 15 mM showed highest effect on enzyme inhibition. **(B)** Huh7 cells were incubated at different time points (6, 12, 24 h) with increasing levels of EP (1–20 mM). WST assays revealed significant dose-dependent reduction of cell proliferation at all time points. **(C1–C7)** Huh7 cells were incubated for 24 h with 1–20 mM EP. Protein analysis showed significant altered expression of proliferation-associated signaling pathways (reduction of PDGFR-β, VEGFR2, VEGF, pERK/ERK, NF-κB; increase of Nrf2). Representative Western Blot images are shown in **(C1)** quantifications **(C2–C7)** are expressed as mean ± S.D. of at least three independent experiments. *P < 0.05, **P < 0.01, ***P < 0.001.

Since Huh7 revealed highest expression and activity of Glo-I in this study, the effects of an inhibition of Glo-I were further analyzed on proliferation and molecular pathways in these cells. Specific Glo-I activity and proliferation were effectively inhibited in a dose-dependent manner. EP at doses of 15 mM resulted in an enzyme activity of 50 ± 13% (*p* < 0.05) compared to no EP (Figure 3A). As expected (14), EP had no influence on protein expression of Glo-I (Supplementary Figure 4). Moreover, EP treatment resulted in a dose-dependent significant reduction of Huh7 proliferation after 6, 12, and 24 h of incubation. After 6 hours, 20 mM EP led to a reduction of cell proliferation to 58 ± 13% (*p* < 0.001), after 12 h to 55 ± 5% (*p* < 0.001) and after 24 h to 57 ± 12% (*p* < 0.001) compared to controls (Figure 3B).

In addition, we analyzed key molecular pathways associated with cell proliferation and migration in HCC (growth factor receptors (PDGFR, VEGFR), its ligands (VEGF), and its downstream cascades (MAP2K/ERK), and transcription factors (NF-κB and Nrf2) (28). Treatment of Huh7 cells with 20 mM EP for 24 h revealed a significantly lower protein expression of PDGFR-β (18 ± 10%, *p* < 0.05), VEGFR2 (46 ± 11%, *p* < 0.01) and its ligand VEGF (61 ± 10%, *p* < 0.01). Moreover, EP resulted in reduced pERK/ERK ratio (62 ± 6%, *p* < 0.01) and NF-κB (44 ± 12%, *p* < 0.05) levels but elevated expression of Nrf2 (243 ± 36%, *p* < 0.01) as compared to the untreated controls (100%; Figures 3C1–C7). Another HCC cell line, HepG2, was used in order to confirm the previous results. In summary, comparable results were found with lower expression of growth factor receptors, downstream cascades and transcription factors by increasing concentrations of EP (Supplementary Figure 2).

### Inhibition of Glo-I by BrBzGSHCp_2_ Also Reduces Proliferation and Related Pathways in Huh7 Cells

**Figure 4:**
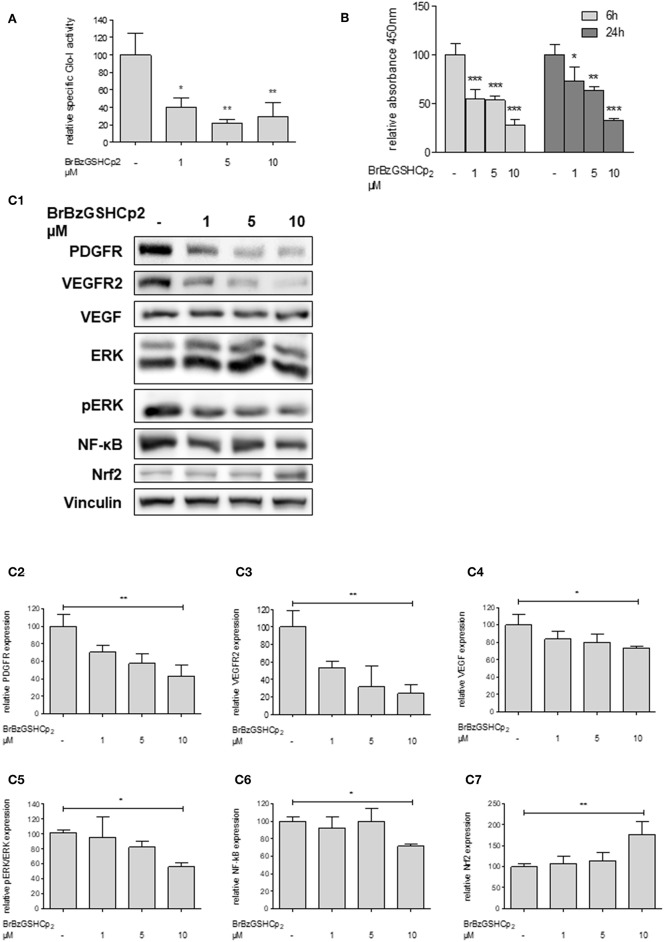
Effect of inhibition of Glo-I by BrBzGSHCp_2_ on proliferation and related signaling pathways in Huh7 cells. **(A)** Specific activity of Glo-I was significantly reduced after 24 h of BrBzGSHCp_2_ treatment in a concentration-dependent manner indicating strong inhibition of enzymatic activity. **(B)** Huh7 cells were incubated at different time points (6, 24 h) with increasing levels of BrBzGSHCp_2_ (1–10 μM). WST assays revealed significant dose-dependent reduction of cell proliferation at each time points. **(C1–C7)** Huh7 cells were incubated for 24 h with 1–10 μM BrBzGSHCp_2_. Protein analysis showed significant influence on proliferation signaling pathways (reduction of PDGFR-β, VEGFR2, VEGF, pERK/ERK, and NF-κB; increase of Nrf2). Representative Western Blot images are shown in **(C1)** quantifications **(C2–C7)** are expressed as mean ± S.D. of at least three independent experiments. *P < 0.05, **P < 0.01, ***P < 0.001.

To confirm that Glo-I inhibition is responsible for the reduced proliferation of HCC cells, another independent inhibitor of Glo-I, S-p-bromobenzylglutathione cyclopentyl diester (BrBzGSHCp_2_) (21), was used. Treatment of Huh7 cells with BrBzGSHCp_2_ for 24 h markedly decreased the specific activity of Glo-I in a dose-dependent manner at concentrations of 1–10 μM (10 μM: 29 ± 16%, control: 100 ± 25%, *p* < 0.01, Figure 4A). Proliferation was also significantly reduced upon incubation of Huh7 cells with BrBzGSHCp_2_ for 6 and 24 h, respectively (24 h 10 μM: 32 ± 2%, control: 100 ± 10%, *p* < 0.001, Figure 4B). In addition, proliferation-related receptors and pathways such as PDGFR-β (43 ± 13%, *p* < 0.01), VEGFR2 (24 ± 10%, *p* < 0.01), VEGF (73 ± 2%, *p* < 0.05), pERK/ERK (56 ± 5%, *p* < 0.05) and NF-κB (72 ± 3%, *p* < 0.01) showed dose-dependent reduced expression after 24 h of treatment with BrBzGSHCp_2_ at up to 10 μM (Figures 4C1–C6). Furthermore, BrBzGSHCp_2_ led to a significant increase of Nrf2 expression (177 ± 30%, *p* < 0.01, Figure 4C7).

### Inhibition of Glo-I Decreases Migration and Colony Formation in Huh7 Cells

**Figure 5:**
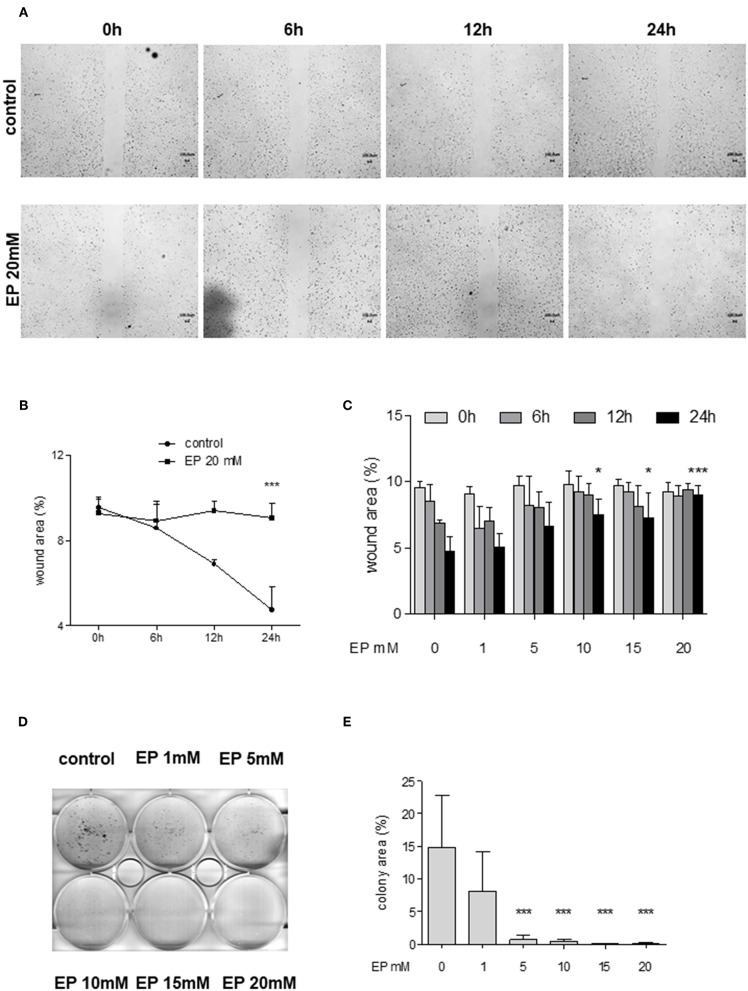
Effect of partial inhibition of Glo-I by EP on migration and colony formation. **(A–C)** Analysis of migration by means of scratch assays. Huh7 cells were seeded in IBIDI dishes and incubated for 24 h (until confluent). After removal of the inlay, cells were treated with increasing amounts of EP (1–20 mM). Images of the wound area were taken at three different time points [6, 12, and 24 h, **(A)**]. Treatment of Huh7 with 20 mM EP showed significant reduction of migration after 24 h, indicated by higher wound area in the EP group **(B)**. **(C)** All measured time points and levels of EP concentrations are shown. Cell migration was significantly inhibited in a dose-dependent manner. **(D,E)** Inhibition of Glo-I resulted in reduced colony formation. Inhibitory effects of EP were assessed using a clonogenic assay. Cells were treated daily with EP (1–20 mM) for 7 days until colonies with >50 cells were seen. Representative images are shown in **(D)**, quantification **(E)** of at least three independent experiments revealed significant reduction of colony formation upon EP-treatment in a dose-dependent manner. Results are expressed as mean ± S.D. *P < 0.05, ***P < 0.001.

In order to examine the effects of Glo-I inhibition on cell migration, scratch assays were performed. Huh7 cells were treated with 1–20 mM EP for 6 to 24 h. The results indicated a significant dose-dependent reduction in cell migration starting at doses of 10 mM EP. After 24 h of incubation, the wound area of controls was significantly reduced (4.6 ± 1.1%) compared to treatment with 20 mM EP (9 ± 1%, *p* < 0.001, Figures 5A–C), indicating a significant reduction in cell migration due to EP.

To analyze colony formation of Huh7, clonogenic assays with Coomassie Blue staining were conducted. After 7 days of incubation with rising concentrations of EP, a significant reduction of colony formation of Huh7 cells was found. Untreated cells revealed more than 50 colonies, with a mean colony area of 15 ± 8% of the well-plate. In contrast, treatment with EP concentrations of 5 mM or higher showed a significant decrease in colony formation (20 mM: 0.7 ± 0.8% of well-plate area, *p* < 0.001, Figures 5D,E). However, cells that were only treated once with EP for 4–24 h and left for another 7 days did not show any significant reduction in colony formation (Supplementary Figures 3A1–B2).

### Sorafenib Increases Glo-I-Expression, -Activity, and Concentrations of MGO

**Figure 6:**
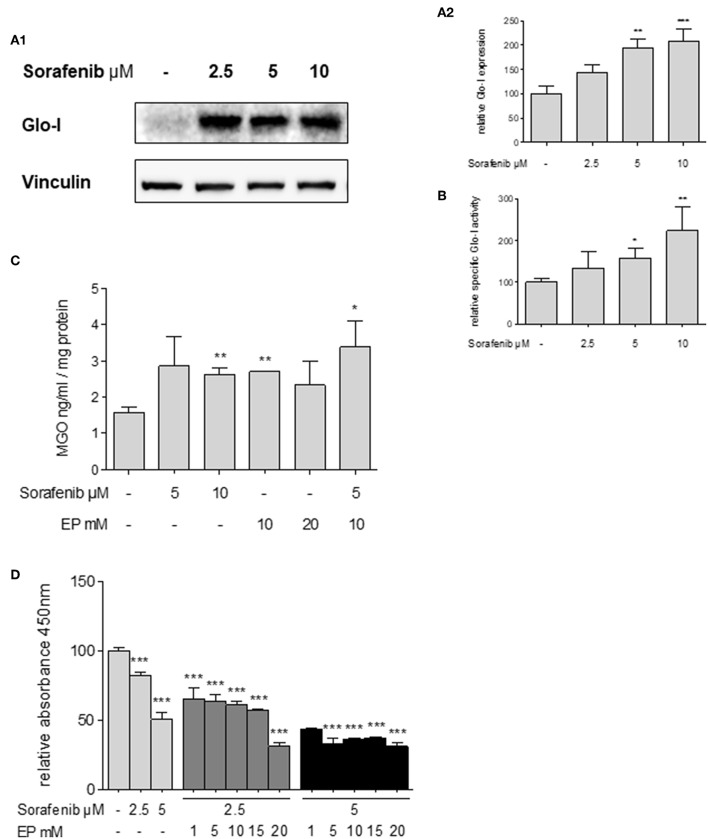
Effects of sorafenib on Glo-I and MGO and the influence of co-treatment with EP. **(A1,A2)** Western Blot analysis of Glo-I expression upon sorafenib treatment in Huh7 cells. Rising levels of sorafenib (2.5–10 μM) increased the protein expression of Glo-I compared to controls. In addition, specific Glo-I activity **(B)** was significantly stimulated after sorafenib treatment in Huh7 cells. **(C)** Effect of sorafenib and EP on MGO levels measured via ELISA. After incubation with sorafenib, production of MGO was significantly increased. Also, EP treatment resulted in elevated MGO-levels. Co-treatment of sorafenib and EP also increases MGO concentrations significantly. **(D)** Influence of sorafenib on proliferation in Huh7 cells measured via WST-assay. Rising concentrations of sorafenib reduced cell proliferation after 24 h incubation. Co-incubation of EP and sorafenib resulted in an additive reduction of cell proliferation compared to incubation with sorafenib or EP alone. Results are expressed as mean ± S.D. of at least three independent experiments. *P < 0.05, **P < 0.01, ***P < 0.001.

Glo-I expression has been linked to multi-drug resistance in cancer chemotherapy in numerous cancers (29). Thus, the effects of sorafenib, a multi-tyrosine kinase inhibitor approved for the therapy of advanced HCC, were studied. Treatment of Huh7 cells with sorafenib led to a significant increase in expression of Glo-I at concentrations of 5 μM (194 ± 18%, *p* < 0.01) and 10 μM (209 ± 25%, *p* < 0.001) compared to controls (100 ± 15%, Figures 6A1,A2). In addition, the specific enzymatic activity of Glo-I was also upregulated at 5 μM (159 ± 23%, *p* < 0.05) and 10 μM (224 ± 58%, *p* < 0.01) doses of sorafenib (Figure 6B).

Sorafenib is known to induce oxidative-stress related cell death in HCC (30). To address if the upregulation of Glo-I in sorafenib treated cells is due to MGO-derived oxidative stress, the effect of sorafenib on MGO levels in Huh7 cells was explored. After 24 h of incubation, MGO levels were significantly increased at 10 μM (2.6 ± 0.2 ng/ml vs. 1.6 ± 0.2 ng/ml, *p* < 0.01) doses of sorafenib (Figure 6C). Interestingly, treatment with doses of 10 mM EP or higher also led to elevated levels of MGO (2.7 ± 0.1 ng/ml, *p* < 0.01). Consequential, co-treatment of EP and sorafenib resulted in a further increase of MGO concentration (3.4 ± 0.7 ng/ml, *p* < 0.05, Figure 6C).

### Inhibition of Glo-I Increases the Sensitivity of Huh7 Cells to Antiproliferative Effects of Sorafenib

To confirm, if inhibition of Glo-I results in a higher susceptibility of HCC cells to the antiproliferative effects of sorafenib treatment (31), cell proliferation was analyzed using a WST-assay. Treatment with 5 μM sorafenib led to a significant reduction in cell proliferation of Huh7 cells (50.4 ± 2.6%, *p* < 0.001, compared to controls, 100 ± 3%, Figure 6D). Moreover, co-treatment of rising concentrations of EP and sorafenib resulted in an additional inhibitory effect on cell proliferation in contrast to either 2.5 μM or 5 μM sorafenib treatment alone (2.5 μM sorafenib + 15 mM EP: 57 ± 1% vs. 2.5 μM sorafenib: 82 ± 1, *p* < 0.001; 5 μM sorafenib + 15 mM EP: 37.16 ± 0.3% vs. 5 μM sorafenib: 50.4 ± 2.6%, *p* < 0.01, Figure 6D). Interestingly, lower doses of sorafenib and higher doses of EP showed a similar decrease in proliferation and vice versa.

## Discussion

The main results of this study show a high expression of Glo-I in human HCC tissues and in more invasive HCC cell lines. For this matter, different HCC cell lines were investigated to show the interaction of Glo-I with key signaling pathways involved in the carcinogenesis of HCC. Inhibiting Glo-I with two distinct inhibitors, led to a significant downregulation of these pathways. In addition, migration, proliferation and colony formation were significantly reduced due to Glo-I inhibition. In contrast, treatment of HCC cells with sorafenib caused a significant upregulation of Glo-I expression and kinetic activity. Co-treatment of cells with Glo-I inhibitors and sorafenib enhanced the anti-proliferative effects, respectively.

Glo-I is highly expressed in HCC, as our and other studies confirm (16, 18). This study reveals a much higher expression of Glo-I in HCC tissues as compared to non-HCC cirrhotic tissue. A low expression of Glo-I in non-HCC cirrhotic tissue was also seen in our previous results (14). An upregulation of Glo-I was also found in many other tumor types (e.g., breast cancer), that have been linked with poor outcome (32). Among the cell lines studied, Glo-I showed highest expression in Huh7 cells which are known to resemble a high-risk HCC group with poor prognosis and early metastasis (33). An upregulation of Glo-I might suggest a certain dependence of invasive cancer cells on MGO-detoxification due to high glycolytic activity. This is confirmed by recently published data showing a knockdown of Hexokinase 2 (HK 2), which marks the first step in glycolysis, to inhibit proliferation in Huh7 more than in HepG2 cells (34). This supports the assumption that Glo-I is favorably upregulated in cells with increasing potential to proliferate and to eventually metastasize. In the present study, proliferation of Huh7 was highly associated with higher Glo-I expression and kinetic activity. In contrast, inhibition of Glo-I reduces proliferation, migration and colony formation in Huh7 cells. This is in line with findings by a study that showed a greater decrease in migration and invasion of Huh7 compared to HepG2, when incubated with MGO solely (35).

Little is known about the downstream signaling following an inhibition of Glo-I in HCC. This study reveals a high interdependence of Glo-I with growth factor receptors (PDGFR-β, VEGFR2 and its ligand VEGF), as well as the downstream signaling via ERK/pERK in HCC. Glo-I inhibition showed a decrease in expression of these receptors and signaling, respectively. This might be explained by an overall enhanced metabolism in cancer cells due to growth factor stimulated proliferation (5). Thus, Glo-I may serve as a detoxifying mechanism in this cross-talk. To what extent Glo-I regulates the expression directly, was not the focus of our study; but many studies have investigated the impact of high MGO levels on tumor growth. Indeed, increasing concentrations of MGO were shown to cause dysfunction of PDGFR-β and impaired ERK activation (36). Another study described a decrease of VEGFR2 expression due to altered MGO concentrations, which was abolished by Glo-I overexpression (37). This was verified by increased MGO levels after EP related Glo-I inhibition. Nevertheless, the role of MGO accumulation in tumor cells remains controversial. Lower levels may contribute to tumor growth, whereas high levels of MGO exert toxic effects (38). Our sorafenib findings are in line with the idea of toxic MGO levels to be associated with downregulated cancer-related pathways. As a result, MGO concentrations were increased in both cases, sorafenib and EP, respectively.

Multi-drug resistance (MDR) is one of the main mechanisms leading to lower treatment response in tumor therapy. That MDR is also involved in therapy failure of sorafenib has been shown in several studies before (39). Glo-I is thought to induce MDR in cancer cells (15). In this context, the present study provides evidence that sorafenib increases the expression and activity of Glo-I. This may seem counterintuitive at first because sorafenib activates Glo-I and decreases proliferation, whereas normally low levels of Glo-I activity would cause less proliferation. One possible explanation is that sorafenib causes cellular cytotoxicity due to MGO induction. In fact, methylglyoxal levels were significantly higher following sorafenib incubation. In this case, higher Glo-I expression after sorafenib treatment may be a result of activated Nrf2, which is the main transcription factor regulating the expression of antioxidant proteins. As a result, Nrf2 increases the expression of Glo-I if stimulated by oxidative stress (40). Therefore, sorafenib may increase the expression of Glo-I via transcription in an oxidative stress and Nrf2 mediated pathway. In fact, sorafenib was shown to induce the expression of Nrf2 in HCC cell lines (41). That sorafenib also decreases proliferation may be due to its inhibitory actions on tyrosine kinases important for cell growth (31). In this study, an elevation of Nrf2 was also seen when Glo-I was inhibited. This strengthens the hypothesis that impaired Glo-I activity causes MGO accumulation and thus oxidative stress. More intriguingly, it also suggests that Glo-I does not influence the expression of Nrf2 directly. Instead, elevated expression of Nrf2 is due to rising concentrations of cellular stress signals such as MGO.

Inhibition of Glo-I sensitizes Huh7 cells to treatment with sorafenib. The anti-proliferative effects of sorafenib were significantly higher, when Glo-I was inhibited. This could be due to the diminished detoxifying effects of Glo-I. Higher concentrations of MGO were detected after co-incubation with EP and sorafenib. This suggests that without the detoxification by Glo-I, MGO levels reach a toxic threshold exerting anti-proliferative and pro-apoptotic mechanisms (42).

This study has some limitations. Instead of a Glo-I knockdown, two independent pharmacologic inhibitors were used. Nevertheless, a pharmacological treatment is closer to a clinical approach than a genetic knockout. For instance, a CRISPR/Cas 9 knockout of Glo-I in Schwann cells did not show elevated MGO nor MGO specific protein modifications due to a compensatory upregulation of aldose reductase (43). The results of our clonogenic assay showed a similar aspect: The daily administration of EP, thus resembling a clinical regimen, decreased colony formation significantly as compared to a single treatment with EP. Furthermore, investigating Glo-I in rodent models is difficult to perform as highlighted by a knockdown of Glo-I in a mouse model that did not affect the level of MGO-derived AGEs in the liver, although Glo-I activity was downregulated (44). A heterozygous Glo-I knockout mice failed to show any difference in Glo-I activity and expression in a variety of tissues, including the liver (45). Recent data also showed conflicting results regarding Glo-I knockdowns in HCC (17, 18). Taking together, Pharmacologic inhibitors may be more promising to investigating immediate or acute changes upon inhibition.

In conclusion, the present study showed the significance of Glo-I in proliferation, migration and colony formation of HCC. Combining sorafenib with Glo-I inhibitors may exert synergistic effects. In this context, decreasing cancer related pathways and inhibiting possible counter-mechanisms, may increase the efficacy of treatment with sorafenib. Yet, further clinical investigations of Glo-I expression and sorafenib response, as well as survival rates are warranted to verify these results.

## Data Availability

The datasets generated for this study are available on request to the corresponding author.

## Author Contributions

MM and MH performed the experiments, analyzed data, and wrote the manuscript. SP gave technical advice. MH and AZ planned the study. MM, MH, CR, and AZ discussed data and revised the manuscript.

### Conflict of Interest Statement

The authors declare that the research was conducted in the absence of any commercial or financial relationships that could be construed as a potential conflict of interest.

## Supplementary Material

Supplemental Figure 1 Glyoxalase system. Glyoxalase I and glyoxalase II comprise the glyoxalase system for detoxification of MGO. Glutathione is necessary as cofactor and is regenerated by Glo-II.

Supplemental Figure 2 Effects of partial inhibition of Glo-I by EP on proliferation-related pathways in HepG2 cells. **(A1,A2)** HepG2 cells were treated with 1–20 mM EP for 24 h. EP treatment resulted in significantly reduced expression of PDGFR-β (20 mM EP: 28 ± 8%, control: 100 ± 16%, *p* < 0.01), VEGFR2 (20 mM EP: 16 ± 10%, control: 100 ± 18, *p* < 0.001), pERK/ERK ratio (20 mM EP: 28 ± 7%, control: 100 ± 24%, *p* < 0.01), and NF-κB (20 mM EP: 58 ± 14%, control: 100 ± 11%, *p* < 0.05). Representative Western Blot images are shown in **(A1)**, quantifications **(A2)** were calculated of at least three independent experiments. Inhibition of specific Glo-I activity by EP is shown in **(B)** (15 mM EP: 52 ± 5%, control: 100 ± 3%, *p* < 0.01). Results are expressed as mean ± S.D. ^*^*p* < 0.05, ^**^*p* < 0.01, ^***^*p* < 0.001.

Supplemental Figure 3 Effect of short-time Glo-I inhibition via EP on colony formation. **(A1–B2)**, Clonogenic assays were performed for 7 days until colonies with >50 cells were seen. Huh7 cells were treated with EP (1–20 mM) only for 4 h **(A1,A2)** or for 24 h **(B1,B2)** to analyze the influence of a short-time inhibition of Glo-I on colony formation. Neither 4 h nor 24 h of treatment revealed significant reduction of colony formation after 7 days. Representative images are shown in **(A1,B1)**, quantifications **(A2,B2)** of at least three independent experiments revealed no significant reduction of colony formation upon EP-treatment. Results are expressed as mean ± S.D.

Supplemental Figure 4 Effect of EP on protein expression of Glo-I. **(A1,A2)** Huh7 cells were treated for 24 h with 1–20 mM EP. Western Blot analysis showed no significant alterations in protein expression of Glo-I. Representative Western Blot images are shown in **(A1)**, quantification **(A2)** was performed of at least three independent experiments. Results are expressed as mean ± S.D.
