# Supplementary material for: Inhibition of Glyoxalase-I Leads to Reduced Proliferation, Migration and Colony Formation, and Enhanced Susceptibility to Sorafenib in Hepatocellular Carcinoma
Source: Front Oncol. 2019 Aug 20;9:785. doi: 10.3389/fonc.2019.00785 (PMC6710403; doi:10.3389/fonc.2019.00785)
Supplement: Supplemental Figure 1 — Glyoxalase system. Glyoxalase I and glyoxalase II comprise the glyoxalase system for detoxification of MGO. Glutathione is necessary as cofactor and is regenerated by Glo-II. [file Presentation_1.PPTX]

## Slide 1
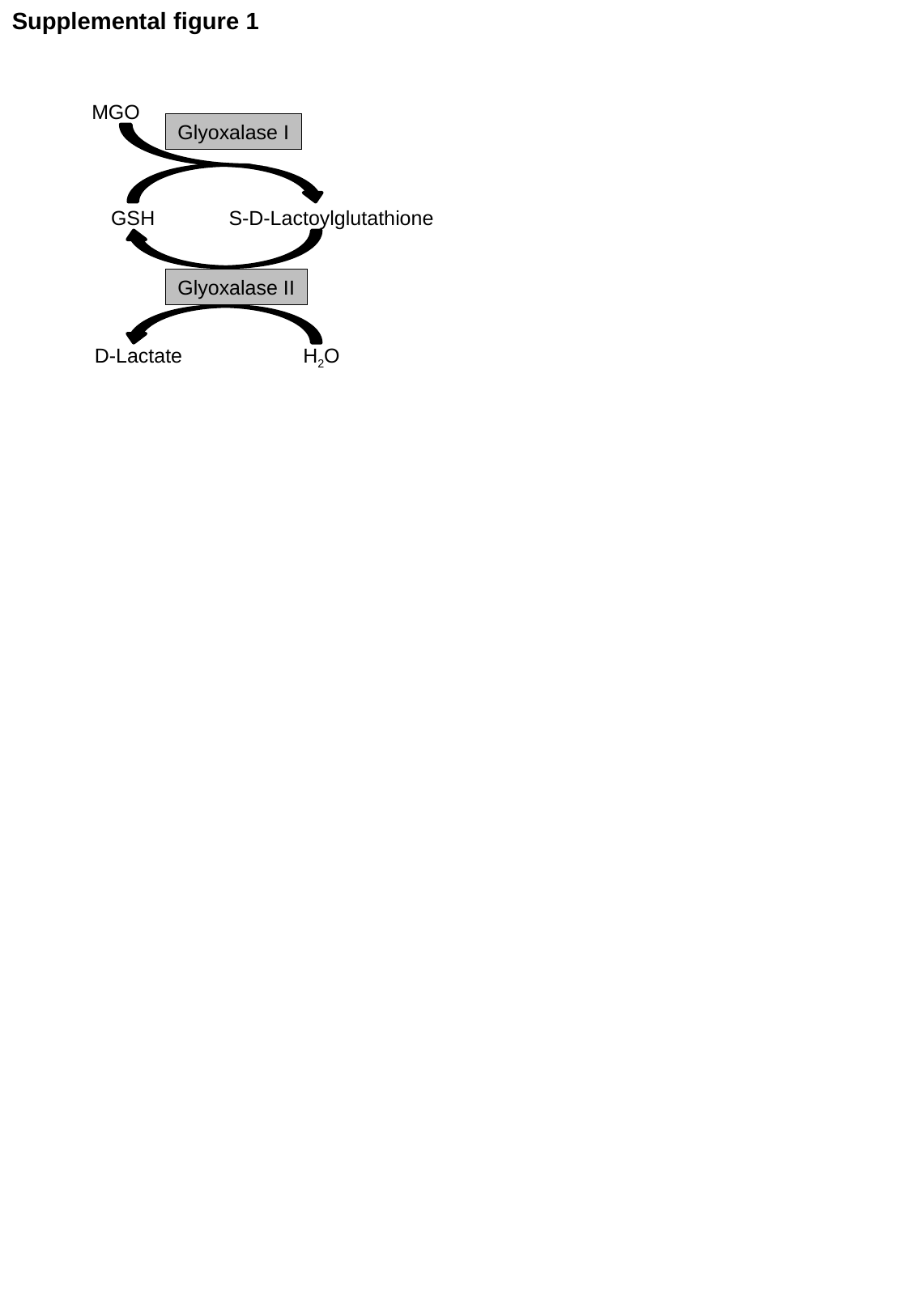

Supplemental figure 1
MGO
Glyoxalase I
GSH
S-D-Lactoylglutathione
Glyoxalase II
D-Lactate
H2O
