# Supplementary figures and images for: Inhibition of Glyoxalase-I Leads to Reduced Proliferation, Migration and Colony Formation, and Enhanced Susceptibility to Sorafenib in Hepatocellular Carcinoma
Source: Front Oncol. 2019 Aug 20;9:785. doi: 10.3389/fonc.2019.00785 (PMC6710403; doi:10.3389/fonc.2019.00785)

## Slide 1
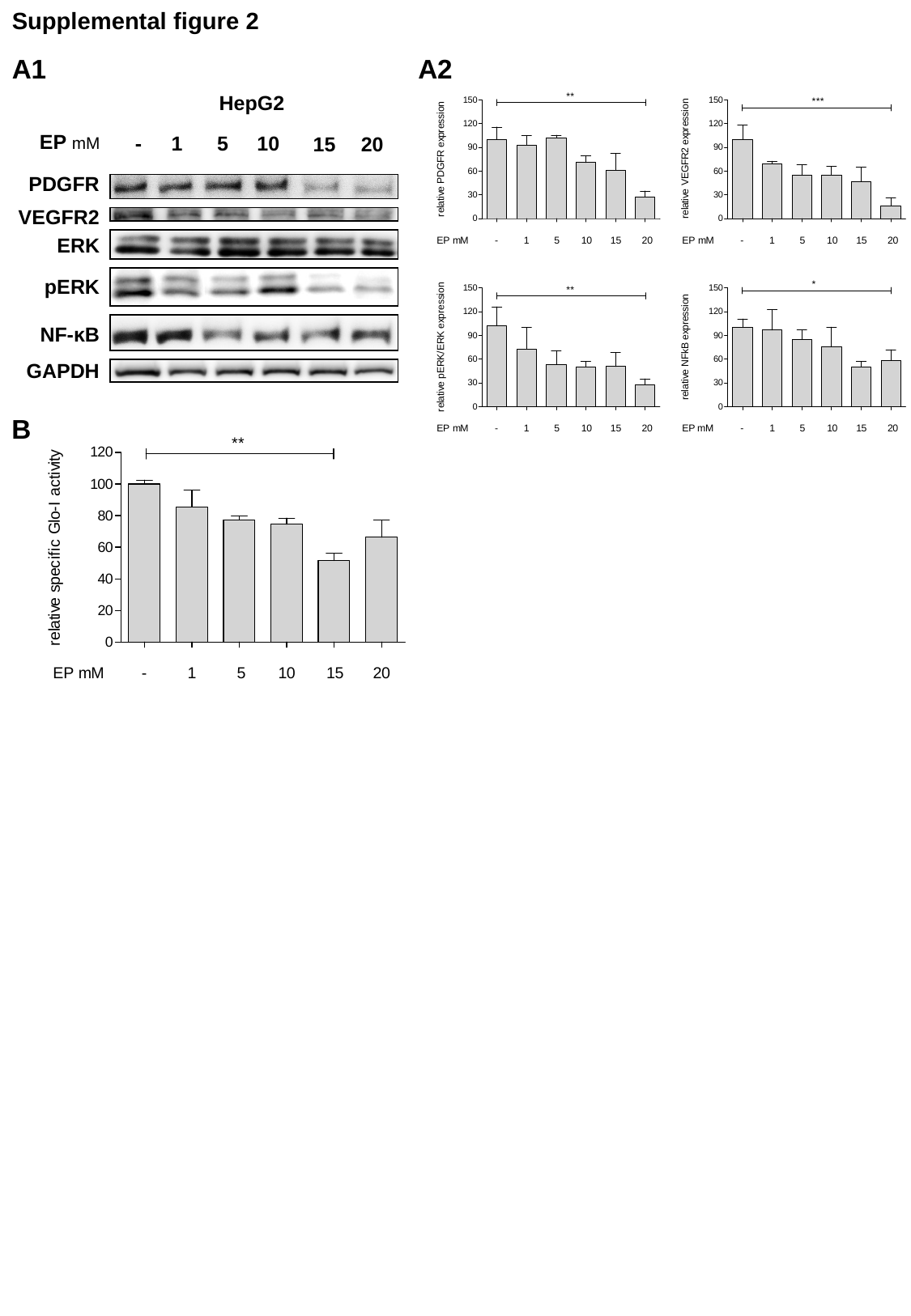

Supplemental figure 2
A1
A2
HepG2
EP mM
-
1
5
10
15
20
PDGFR
VEGFR2
ERK
pERK
NF-ĸB
GAPDH
B

Supplement: Supplemental Figure 2 — Effects of partial inhibition of Glo-I by EP on proliferation-related pathways in HepG2 cells. (A1,A2) HepG2 cells were treated with 1–20 mM EP for 24 h. EP treatment resulted in significantly reduced expression of PDGFR-β (20 mM EP: 28 ± 8%, control: 100 ± 16%, p < 0.01), VEGFR2 (20 mM EP: 16 ± 10%, control: 100 ± 18, p < 0.001), pERK/ERK ratio (20 mM EP: 28 ± 7%, control: 100 ± 24%, p < 0.01), and NF-κB (20 mM EP: 58 ± 14%, control: 100 ± 11%, p < 0.05). Representative Western Blot images are shown in (A1), quantifications (A2) were calculated of at least three independent experiments. Inhibition of specific Glo-I activity by EP is shown in (B) (15 mM EP: 52 ± 5%, control: 100 ± 3%, p < 0.01). Results are expressed as mean ± S.D. *p < 0.05, **p < 0.01, ***p < 0.001. [file Presentation_2.PPTX]

## Slide 1
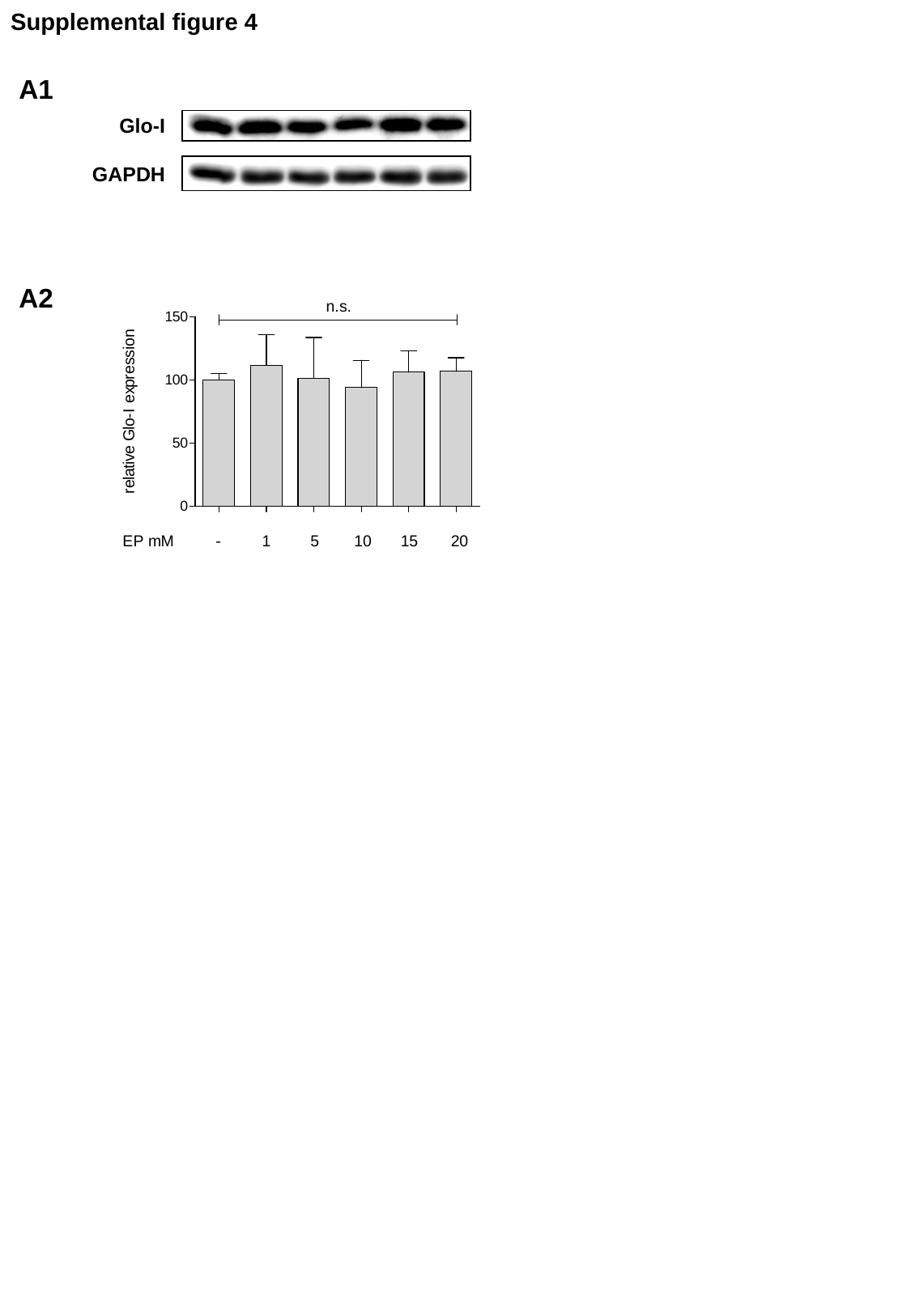

Supplemental figure 4
A1
Glo-I
GAPDH
A2

Supplement: Supplemental Figure 4 — Effect of EP on protein expression of Glo-I. (A1,A2) Huh7 cells were treated for 24 h with 1–20 mM EP. Western Blot analysis showed no significant alterations in protein expression of Glo-I. Representative Western Blot images are shown in (A1), quantification (A2) was performed of at least three independent experiments. Results are expressed as mean ± S.D. [file Presentation_4.PPTX]
