# Supplementary material for: Inhibition of Glyoxalase-I Leads to Reduced Proliferation, Migration and Colony Formation, and Enhanced Susceptibility to Sorafenib in Hepatocellular Carcinoma
Source: Front Oncol. 2019 Aug 20;9:785. doi: 10.3389/fonc.2019.00785 (PMC6710403; doi:10.3389/fonc.2019.00785)
Supplement: Supplemental Figure 3 — Effect of short-time Glo-I inhibition via EP on colony formation. (A1–B2), Clonogenic assays were performed for 7 days until colonies with >50 cells were seen. Huh7 cells were treated with EP (1–20 mM) only for 4 h (A1,A2) or for 24 h (B1,B2) to analyze the influence of a short-time inhibition of Glo-I on colony formation. Neither 4 h nor 24 h of treatment revealed significant reduction of colony formation after 7 days. Representative images are shown in (A1,B1), quantifications (A2,B2) of at least three independent experiments revealed no significant reduction of colony formation upon EP-treatment. Results are expressed as mean ± S.D. [file Presentation_3.PPTX]

## Slide 1
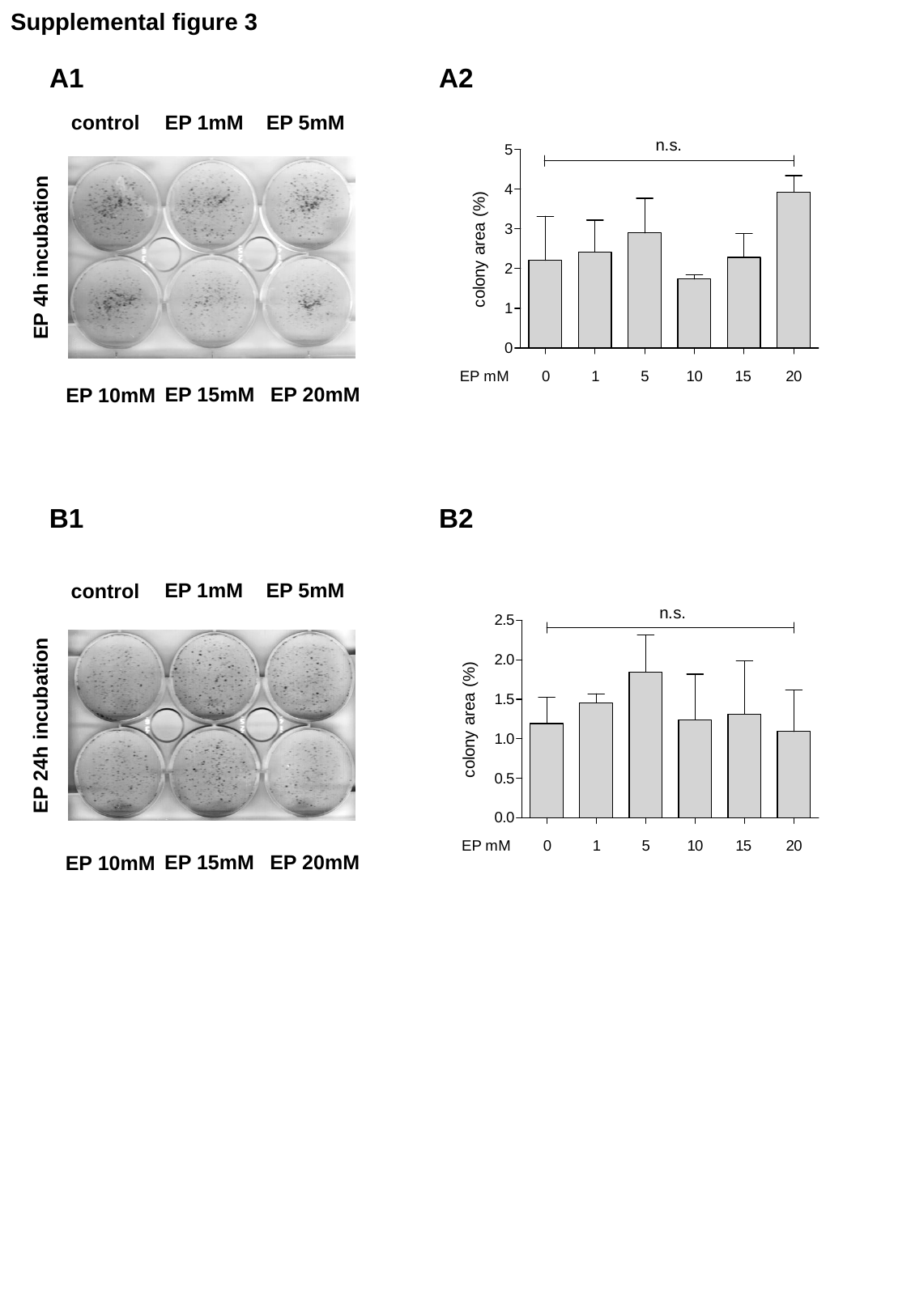

Supplemental figure 3
A1
A2
EP 1mM
EP 5mM
control
EP 4h incubation
EP 15mM
EP 20mM
EP 10mM
B1
B2
EP 1mM
EP 5mM
control
EP 24h incubation
EP 15mM
EP 20mM
EP 10mM
